# Perry's Remarks in Conjunction with Bonwill on Amalgams and Anticipation, Etc.

**Published:** 1888-10

**Authors:** W. G. A. Bonwill

**Affiliations:** Philadelphia, Pa.


ARTICLE IV.

### PERRY'S REMARKS IN CONJUNCTION WITH BONWILL ON AMALGAMS AND ANTICIPATION, ETC.

DR. W. G. A. BONWILL, PHILADELPHIA, PA.

*Editor Items of Interest*:

I must call your attention to May number of *Items of Interest*, where you have made extracts from the *Cosmos* of my article on "Amalgams," and attached the remarks of Dr. I. G. Perry, from a subsequent number of *Cosmos*, where I am made to say that I had abandoned *self-cleansing surfaces* or separations to preserve teeth from further decay without filling, regretting that I had ever done it. You are so very careful and correct in your selections I cannot blame you very much for this serious mistake.

Now I should not be Bonwill, unless I had the liberty of changing my views, my practice, or my instruments whenever I please. Circumstances meet me every day where I am compelled to vary from the beaten course or fail. No one can in the practice of dentistry chain himself to an absolute system in each and every case. No better field for ingenuity looms up before the wide-awake operator. Any one who will follow my past history must admit that I have been constantly on the change, cutting off here and adding there, etc. I am able to do it and proud that I have the independence to do so!

Have I changed at all in my views of the practice of anticipating decay in human teeth? So far as the theory is concerned, I say no! Have I changed in its application to the age of patient and the character of the teeth, and the caries met with? Yes! Thirty years ago, teeth were very different in constitution than now. Then, a separation after decay had commenced could be checked. Fifteen years ago, I could anticipate decay by the use of special instruments in my dental engine, and in two-thirds of the bicuspids and molars I could save from further caries. The superior or inferior incisors I could save seven-eighths at least. To-day, or during the past five years, I am not able even to preserve a tooth from further ravage of caries by Arthur's or any other plan where decay has once commenced, without filling with some material.

In many cases, when I have attempted to *anticipate*, believing there was but a trifle of caries, I have been baffled; for as soon as the separation has been made, I detect a change of color, but the surface has not yet given away sufficiently to allow me to get into the substance changed, such fine exploring needles as I use. A small bur in the engine very soon tells the tale that a hole of slight diameter, but deep in proportion, is actually there. The exploring needle will not pass into the changed tissue. It can be detected only by the educated eye, and touch, and by the aid of a dried surface, with a strong light.

So soon do teeth commence to decay after contact, that with young patients, when they are not careful in cleansing, and the temporary teeth still in position with their broad flat surfaces in contact with the coming permanent tooth, that it is almost impossible to anticipate decay except in the temporary set, when you can commence as early as the third year, and in the cases of the present generation in the past eight years. Separation of any kind cannot insure success, for decay has gone beyond the boundary and filling must be done. The poor constitution of teeth generally found in large cities offer no hope for anticipation or separation when decay has shown itself.

To commence so early as the third year for the temporary and the twelth year for the permanent set, except in the incisors, will not do unless you can have the perfect consent of patient and their complete obedience in submitting to the unpleasantness of the engine. The present average child is so nervous over the treatment of the teeth that they are very difficult to manage, and parents no longer have their progeny under control, nor have they the powers of endurance requisite for a healthy life.

What are we to expect in the future from any treatment looking to the anticipation of decay, by cutting or by filling when we find the temporary molar on its distal surface, where nothing but the gum is in contact, running to decay, shortly after it is in position? Also the first permanent molar on its distal surface, when the second molar is nowhere near? The imperfect calcification of the tooth with the class of soft food now used and the imperfect cleansing, and the immense amount of saccharin that daily and hourly enter the mouth and clog on the surfaces of the teeth, make the problem harder to solve each coming year. This, to say nothing of the class of operations performed by most dentists; the want of method in laying out their work and systematically taking the teeth to be treated that most need it; the want of foresight in commencing where they should have completed the operations; the general lack of engineering skill in mapping out the whole thing in advance, etc., all warn us that we lack that peculiar training given to the mechanical and civil engineer of prospecting the work and taking in the whole thing before we commence an individual act.

Then, sir, I still hold to the principle of cutting for anticipation in both the temporary and permanent sets. Indiscriminately, however, it cannot be done. We must have surroundings or we cannot carry out any principle. The lack of constitution then, in the present tooth, forbids the general practice of anticipation or separation. That done by me years ago when substance was better, I know did its work, when I am confident decay would have occurred inevitably. This I have proven so often by anticipation on the most unfavorable side of the mouth and allowing the opposite side to remain in normal contact.

The judicious extraction of the first permanent molars in but few cases saves the remaining teeth from caries. Even where the bicuspids have separated from each other, I have found them decaying where no cause of contact before had predisposed. I have occasion to see every year, a tooth—first inferior bicuspid left—that thirty-four years ago I removed the entire enamel and left it a perfect pyramidal cone which could not allow a particle of food at any point to accumulate thereon. It remained perfect till two years ago, when I found on its polished surface, where most exposed to food and the brush, decay treacherously coming. I filled it with amalgam, and in another year, at a point near the apex of the cone, I found another carious spot. What but the constitution of the tooth undergoing a change in its very substance at the age of fifty-two could have produced it? All the upper teeth were lost at twenty-two. All the lower I have preserved for thirty-four years, when, at the time I first commenced their treatment in 1854, scarcely an inferior incisor but had caries deep enough to demand a filling. But notwithstanding their poor structure, only two pulps have been treated and not a tooth lost in the inferior jaw, and yet this one inferior bicuspid that resisted over thirty years at last decayed. There were several others I cut at the same time that did not afterward decay.

Here is a case that had poor teeth at that early age, that, from cutting with a chisel and smoothing with a file and stone—no engine—resisted decay and gave way only on one cut surface. He kept his teeth scrupulously clean and came once a year for treatment. Where I did not cut, not an proximal surface but had to be filled.

A word as to self-cleansing surfaces after filling. For ten or twelve years, I have seldom made them of the boldest character and wide at the cervix; but contoured by cutting, and, by the use of gutta-percha, widening or pressing the teeth apart from mastication on it.

To sum up.—Cutting the surface of any tooth is legitimate and correct practice. It can and should be done in many cases. It cannot be done as a broad gage rule where the average dentist has to do it.

That hundreds of teeth can be saven; and, if decay in the future takes place, there is not so much tooth substance gone. In the incisors, even if poor, I fail not to do it. I expect to do it whenever I believe it prudent. For the ul timate safety of the human teeth, nothing save a change to a more primitive diet, and the proper use of our teeth and observance of hygienic laws will ever give us immunity from caries.—*Items of Interest.*

